# Fluorescence and antioxidant activity of heterologous expression of phycocyanin and allophycocyanin from *Arthrospira platensis*

**DOI:** 10.3389/fnut.2023.1127422

**Published:** 2023-02-20

**Authors:** Meng-hui Shang, Jian-fei Sun, Ying Bi, Xiao-ting Xu, Xiao-nan Zang

**Affiliations:** Key Laboratory of Marine Genetics and Breeding, Ministry of Education, Ocean University of China, Qingdao, China

**Keywords:** phycocyanin, allophycocyanin, recombinant expression, fluorescence activity, antioxidant, *Arthrospira platensis*

## Abstract

Phycocyanin and allophycocyanin are important active substances in *Arthrospira platensis*, because of their fluorescent characteristic and antioxidant capacity. In order to solve the problem of insufficient production and inconvenient modification of natural protein, recombinant expression was performed and the fluorescence activity and antioxidant activity was analyzed to meet the demand for phycocyanin and allophycocyanin. A total of seven recombinant strains were constructed in this study, including individual phycocyanin or allophycocyanin, co-expression of phycocyanin-allophycocyanin, and their co-expression with chromophore, and the expression strain for individual chromophore. Different molecular weights of phycocyanin and allophycocyanin were detected in the recombinant strains, which indicated the different polymers expressed. Through mass spectrometry identification, phycocyanin and allophycocyanin may form a dimer of 66 kDa and a polymer of 300 kDa. The results of fluorescence detection showed that phycocyanin and allophycocyanin combined with phycocyanobilin to show fluorescence activity. The fluorescence peak of recombinant phycocyanin was mainly concentrated at 640 nm, which was similar to natural phycocyanin, the fluorescence peak of purified recombinant allophycocyanin was at about 642 nm. The fluorescence peak of the co-expressed recombinant phycocyanin-allophycocyanin is located at 640 nm, and the fluorescence intensity is between the recombinant phycocyanin and the recombinant allophycocyanin. After purification, the fluorescence peak of the recombinant phycocyanin is more concentrated and the fluorescence intensity is higher, which is about 1.3 times of recombinant phycocyanin-allophycocyanin, 2.8 times of recombinant allophycocyanin, indicating that phycocyanin may be more suitable to be used as fluorescence probe in medicine. The antioxidant capacity was measured by using total antioxidant capacity (T-AOC) and DPPH (2,2'-diphenyl-1-triphenylhydrazino) free radical scavenging method, and the recombinant phycobiliprotein showed antioxidant activity. Phycocyanobilin also has certain antioxidant activity and could enhance the antioxidant activity of phycobiliprotein to a certain extent. Recombinant phycocyanin-allophycocyanin polymer has stronger T-AOC, which is about 1.17–2.25 times that of the other five recombinant proteins. And recombinant phycocyanin has stronger DPPH antioxidant activity, which is about 1.2–2.5 times that of the other five recombinant proteins. This study laid the foundation for the application of recombinant phycocyanin and allophycocyanin in medical detection and drug development.

## 1. Introduction

*Arthrospira platensis* is a filamentous prokaryotic cyanobacteria, which is rich in nutrients and contains all the essential amino acids for the human body. Its protein content is high, accounting for 60–71% of the dry weight of cells (1, 2). Phycobiliprotein is its most important protein, which accounts for up to 20% of the dry weight of *Arthrospira*. Correspondingly, phycobiliproteins account for almost 1/3 of the total protein of the algae (3). They are not only the main proteins in phycobilisomes, and play an important light-harvesting function in photosynthesis, but also have important physiological functions such as anti-oxidation and anti-inflammation, and are widely used in medicine, food, and other industries (4, 5).

Phycobiliproteins are a class of brightly colored water-soluble proteins. Due to the presence of linear tetrapyrrole structures, they show different color characteristics, so they are considered as natural sources of pigments and are used as natural dyes in food and cosmetics (6). Compared with chemically synthesized fluorescent dyes, phycobiliproteins emit strong fluorescent signals due to their relatively high fluorescence quantum yield and extinction coefficient. Because their fluorescence is not quenched by biomolecules, phycobiliproteins can be used as valuable fluorescent labels in many applications. Phycobiliproteins conjugated to biomolecules such as immunoglobulin or streptavidin have achieved great success in flow cytometry, fluorescence-activated cell sorting (FACS), histochemistry, imaging, and detection of reactive oxygen species. At present, phycoerythrin and allophycocyanin are commonly used in the market because of their fluorescent activity (7–9). Phycobiliproteins also have the properties of removing excessive reactive oxygen species in the body and increasing the activity of antioxidant-related enzymes (10). These mechanisms may eliminate the imbalance between the body's oxidative system and antioxidant system caused by oxidative stress. Therefore, phycocyanin has the potential to treat various diseases caused by oxidative stress. Phycobiliproteins in *Arthrospira* are mainly composed of phycocyanin and allophycocyanin. Although phycocyanin and allophycocyanin have the same chromophore—phycocyanobilin (PCB), their optical properties and physiological functions are different.

Phycocyanin is a component of the rod complex of phycobilisomes. Phycocyanin is a heterodimer composed of two polypeptides, α subunit and β subunit, and generally exists in the form of a hexamer (αβ)_6_. The combination of phycocyanin and phycocyanobilin can exhibit optical activity. Phycocyanin absorbs visible light at about 615–640 nm, with an absorption peak at 620 nm, and emits fluorescence at about 640 nm. Due to the different functional properties of phycocyanin, it plays a very important role in practical applications. The main applications are as follows: First, as a natural food additive and pigment (11, 12). In recent years, due to the safety and health problems of artificial pigments, the application of natural pigments in food and medicine has attracted more and more attention. The natural phycocyanin extracted from *Arthrospira* is a brightly colored water-soluble protein that is widely used as a natural blue colorant in the pharmaceutical, cosmetic and food industries. In addition, the effects of phycocyanin on antioxidation (13–15), anti-inflammation (16–19), anti-cancer (20–22), neuroprotection (23), and liver protection (24) have been verified in many ways. In 1999, Rimbau proposed that phycocyanin might be used as a drug ingredient to treat neurodegenerative diseases induced by oxidative stress, such as Parkinson's disease and Alzheimer's disease (25). Bharathiraja proposed that phycocyanin can selectively remove cancer cells through PDT (photodynamic therapy) (26). In addition, due to the fluorescent properties of phycocyanin at specific wavelengths, it is also used as a fluorescent marker for diagnosis and medical treatment (27, 28).

Allophycocyanin (APC) is a phycobiliprotein located in the core complex of phycobilisomes. It has good solubility and stable physical properties. It mainly plays the role of energy transfer in phycobilisomes. The isolated and purified natural allophycocyanin contains α and β subunits, usually in the form of trimer (αβ)_3_. Allophycocyanin also shows optical activity by combining phycocyanobilin as its chromophore. Allophycocyanin absorbs visible light at about 650–655 nm, with an absorption peak at 650 nm, and emits fluorescence at about 660 nm. Allophycocyanin has a blue-green color feature and can be used as a pigment and nutrient in the food industry. In addition, allophycocyanin has also been shown to have antioxidant and anti-cancer effects (29). Qin's research found that apo-allophycocyanin was involved in the antioxidant and free radical scavenging activity of phycocyanin, and its antioxidant activity may be partly related to the anti-tumor effect of recombinant allophycocyanin (30). Shih's experiments showed that allophycocyanin purified in cyanobacteria had activity against enterovirus type 71 (31). In addition, the directional recombinant allophycocyanin trimer can be used as an optical sensitization material. Studies have shown that the fusion protein of streptavidin and allophycocyanin α subunit (ApcA) is a fluorescent protein that can bind to biotin. This fusion protein (SLA) can be used as a fluorescent marker for immunofluorescence detection, and this is the first report of serial expression of fluorescent proteins for immunofluorescence analysis (32).

Phycocyanin and allophycocyanin have a wide range of uses in medicine, health, etc., and their application potential is huge, but the availability of natural phycocyanin and allophycocyanin is limited. Because allophycocyanin and phycocyanin have similar properties and are difficult to separate, traditional extraction methods can only extract 50–60% of the total allophycocyanin in algae, and it is difficult to obtain higher purity allophycocyanin (33). However, genetic engineering recombinant expression can obtain a large number of products, and it is convenient to modify the products according to the requirements of the application.

At present, there have been many reports on the expression of phycocyanin and allophycocyanin from different species of cyanobacteria, but they mainly focus on exploring the activity and function of individual phycocyanin or allophycocyanin (34, 35). The study of co-recombination expression has not been reported yet. In *Arthrospira*, phycocyanin and allophycocyanin together constitute the main body of phycobilisomes, which exert optical activity and other physiological functions, and there may be synergy or functional complementarity between them. Therefore, this study intends to co-express phycocyanin and allophycocyanin to provide a basis for better application of phycobiliproteins in medicine and food.

Our previous researches have cloned the apo-phycocyanin gene *apo-pcBA* and the apo-allophycocyanin gene *apo-apcAB*, ferredoxin oxidoreductase gene (*pcyA*), heme oxidase gene (*ho*), and chromophore lyase genes *cpcU, cpcS, cpcT, cpcE*, and *cpcF* that catalyze the combination of phycocyanobilin and apo-phycobiliprotein from *Arthrospira platensis* FACHB314. On this basis, this study constructed a heterologous co-expression strain of phycocyanin and allophycocyanin, and detected the fluorescence activity and antioxidant activity of the recombinant phycocyanin, allophycocyanin and their polymer. It lays the foundation for obtaining phycobiliprotein components with biological functions to meet people's needs for phycobiliprotein.

## 2. Materials and methods

### 2.1. Strains and genes

*Escherichia coli* BL21 (DE3) for transformation was obtained from Tsingke Biotechnology (China). Expression vectors pACYCDuet-1 and pETDuet-1 were procured from Novagen (Germany).

Phycocyanin genes consists of *pcA* and *pcB* (DQ406671.1), and allophycocyanin genes include *apcA* and *apcB* (HQ828097.1). These genes were cloned from *A. platensis* FACHB314.

The genes related to synthesize phycocyanobilin (PCB) include heme oxygenase gene *ho* (WP_006617685.1) and phycocyanobilin-ferredoxin oxidoreductase gene *pcyA* (WP_006621708.1). The chromophore lyase genes include *cpcU* (AMW31400), *cpcS* (AMW26792.1), *cpcT* (AMW27064.1), *cpcE* (AHA14838.1), and *cpcF* (AHA14838.1) were cloned from *A. platensis*. FACHB314 to catalyze the binding of phycocyanobilin (PCB) to phycocyanin and allophycocyanin.

### 2.2. Construction of recombinant strains

The pACYCDuet-1 Vector was digested with restriction endonucleases (Thermo Fisher Scientific) *Bam*HI and *Sal*I, and then the genes *pcBA* and *apcAB* were respectively connected to the linear vector with T4 ligase (TransGen Biotech) to obtain plasmids pACYCDuet-*pcBA* and pACYCDuet-*apcAB*. The plasmid pACYCDuet*-pcBA* was then digested with restriction endonucleases *Nde*I and *Xho*I, and the gene *apcAB* was connected to it to obtain the plasmid pACYCDuet-*pcBA-apcAB*. The above three plasmids were respectively transformed into *E. coli* BL21 (DE3) to obtain a strain expressing phycocyanin (*E. coli* PC), a strain expressing allophycocyanin (*E. coli* APC), and a strain expressing both phycocyanin and allophycocyanin (*E. coli* PC-APC).

The genes *ho* (digested by *Bam*HI and *Sac*I), *pcyA* (digested by *Sac*I and *Pst*I) were inserted into the pETDuet-1 Vector to obtain the plasmid pETDuet-*ho-pcyA*, and transformed into *E. coli* BL21 (DE3) to obtain the strain *E. coli* HP.

Then the chromophore lyase genes *cpcU* (digested by *Pst*I and *Sal*I), *cpcS* (digested by *Sal*I and *Hin*dIII), *cpcT* (digested by *Hin*dIII and *Not*I), *cpcE* (digested by *Nde*I and *Aat*II), *cpcF* (digested by *Aat*II and *Kpn*I) were inserted into the plasmid pETDuet-*ho*-*pcyA* to obtain the plasmid pETDuet-*ho*-*pcyA*-*cpcU*-*cpcS*-*cpcT*-*cpcE*-*cpcF*.

The three strains *E. coli* PC, *E. coli* APC, and *E. coli* PC-APC were transformed with the plasmid pETDuet*-ho-pcyA-cpcU-cpcS-cpcT-cpcE-cpcF* respectively to obtain three kinds of double plasmid expression strains *E. coli* PC-APC-HPUSTEF, *E. coli* PC-HPUSTEF, and *E. coli* APC-HPUSTEF.

### 2.3. Recombinant proteins expression

The constructed *E. coli* strains were induced to express, and *E. coli* BL21 was used as a control. The strains were inoculated into LB liquid medium containing corresponding antibiotics (final concentration: chloramphenicol 34 μg/mL, ampicillin 100 μg/mL), cultured at 37°C, 200 rpm until OD≈0.6, then added IPTG (The final concentration of double-plasmid strains was 2 mM, and the final concentration of single-plasmid strains was 1 mM), at 23°C, induced expression for 12 h. After the induction, the bacteria were collected by centrifugation at 4,000 × g for 20 min, resuspended and washed with 0.9% NaCl solution, centrifuged at 10,000 × g for 10 min, resuspended the bacteria with phosphate buffer (0.7 M, pH = 7), and performed ultrasonic disruption, 300 W, 16 min (broken 5 S, intermittent 2.5 S), and finally centrifuged at 10,000 × g for 20 min, and the supernatant total protein solution was collected for subsequent experiments.

### 2.4. Protein purification

The total protein solution was fully combined with Ni-NTA filler (MDBio, China) at a ratio of 4:1, and then the unbound impurities were washed with 5–10 times column volume of Wash Buffer (50 mM PBS, 0.3 M NaCl, 20 mM imidazole). The protein was eluted until the pH or conductivity was balanced, and finally the target protein was eluted with 5–10 times column volume Elution Buffer (50 mM PBS, 0.3 M NaCl, 500 mM imidazole) to obtain a purified target protein solution.

### 2.5. Polyacrylamide gel electrophoresis and Western blot analysis

SDS-PAGE (sodium dodecyl sulfate polyacrylamide gel electrophoresis) was prepared using polyacrylamide gel premix (MDBio, China), with 5% stacking gel and 15% separating gel. After electrophoresis, the gel was stained with Coomassie Brilliant Blue R250 solution or transferred to polyvinylidene fluoride membrane for Western blot detection.

Native-PAGE (native polyacrylamide gel electrophoresis) was prepared using polyacrylamide gel premix (MDBio, China), with a stacking gel of 6% and a separating gel of 8%. After electrophoresis, it was stained with Coomassie Brilliant Blue R250 solution.

In western blot detection, to detect the protein expression, the primary antibody was histidine-tag antibody (Sangon Biotech, China), secondary antibody peroxidase-conjugated goat anti-mouse IgG (Sangon Biotech, China); and to specifically detect the target phycobiliprotein, the primary antibody was phycocyanin and allophycocyanin specific antibody (Sino Biological Inc., China), and the secondary antibody was peroxidase-conjugated goat anti-rabbit IgG (Sangon Biotech, China). After hybridization, DAB solution (MDBio, China) was used for color development.

### 2.6. Mass spectrometric detection

The target band detected in Native-PAGE was excised and detected by mass spectrometry. Mass spectrometry was performed using a Triple TOF 5,600 + system (AB SCIEX) combined with a nanoliter spray III ion source (AB SCIEX, USA). The scanning method was the information-dependent acquisition mode (IDA, Information Dependent Analysis), the scanning time of the first-level TOF-MS single map was 250 ms, and a maximum of 26 charges of 2+ to 5+ were collected in each IDA cycle. The secondary spectrum with a second count >200 cps, the accumulation time of each secondary spectrum was 80 ms. Each cycle time was fixed at 2.5 s, the collision cell energy was set for collision-induced dissociation (CID) of all precursor ions, and the dynamic exclusion was set at 3 s. In order to further judge whether there was a polymer of the target protein. The experiment was completed by Sangon Biotech (Shanghai) Co. Ltd. (Sangon Biotech).

### 2.7. Fluorescence emission spectra analysis

Fluorescence detection of the samples was performed using a HITACHI F-4600 fluorescence spectrophotometer. In total 2 mL of the sample solution was added into a cuvette, and then was put into the fluorescence spectrophotometer. The fluorescence emission spectrum of the sample was measured at an excitation wavelength of 580 nm. Detection index setting: the slit width was 10.0 nm, and the scanning speed was 1,200 nm/min.

### 2.8. Antioxidant analysis

The total antioxidant capacity (T-AOC) and DPPH free radical scavenging capacity were tested using the kits (Nanjing Jiancheng Bioengineering Institute, Nanjing, China).

#### 2.8.1. Detection of total antioxidant capacity

The antioxidant substances in the sample can reduce Fe^3+^ to Fe^2+^, which can form a stable complex with phenanthroline substances. The absorbance at 520 nm wavelength reflect the level of antioxidant capacity.

Definition: At 37°C, every milligram of protein per minute, so that the absorbance (OD) value of the reaction system increases by 0.01, was a total antioxidant capacity unit (U). As shown in the formula (1).


(1) $$\begin{array}{l} U/\left(mgprot\right)=\frac{\left(A_{1}-A_{2}\right)}{0.01}÷T\times \frac{V_{1}}{V_{2}}÷Cpr \end{array}$$

T: Reaction time: 30 min. A_1_: Absorbance of experimental group. A_2_: Absorbance of control group. V_1_: Total volume of reaction system. V_2_: volume of sampling. Cpr: concentration of protein. mgprot/ml (prot refers to the protein).

#### 2.8.2. Detection of DPPH free radical scavenging ability

DPPH free radical has a single electron and its alcohol solution is purple with a strong absorption at 517 nm. When there was a free radical scavenger, its absorption gradually disappears due to its single-electron pairing. The lighter the color, the lower the absorbance. And then the DPPH scavenging ability in the sample can be quantitatively analyzed.

Definition: The DPPH free radical scavenging ability of the sample is represented by the amount equivalent to the antioxidant Trolox calculated from the standard curve. DPPH free radical scavenging rate as shown in the formula (2) and (3).


(2) $$\begin{array}{l} \text{Sample DPPH free radical scavenging rate}\left(\%\right)\;=\;[1-\left(A_{1}-A_{2}\right)\\ \;÷\;A_{3}]\;\times \;100\%\; \end{array}$$

(3) $$\begin{array}{l} \text{Standard curve DPPH free radical scavenging ​​​​​​​​​​​​​​​​​​​​​​​​​​​​​​}\\ \text{ ​rate }\left(\%\right)=\left[1-A(S)÷A(S_{0})\right]\\ \;\times \;100\% \end{array}$$

A_1_: Absorbance of experimental group. A_2_: Absorbance of control group. A_3_: Absorbance of blank control. S: Each concentration of standard tube. S_0_: Standard tube with a concentration of 0.

### 2.9. Statistics and analysis of data

All experiments were set up with 3 parallel samples, each sample was tested for 3 replicates. Statistical analysis was conducted using IBM SPSS Statistics (Version 22). If the *P-*value was less than 0.05, the difference was statistically significant, on the contrary, the difference was not significant.

## 3. Results

### 3.1. Construction of recombinant strains

**Figure 1:**
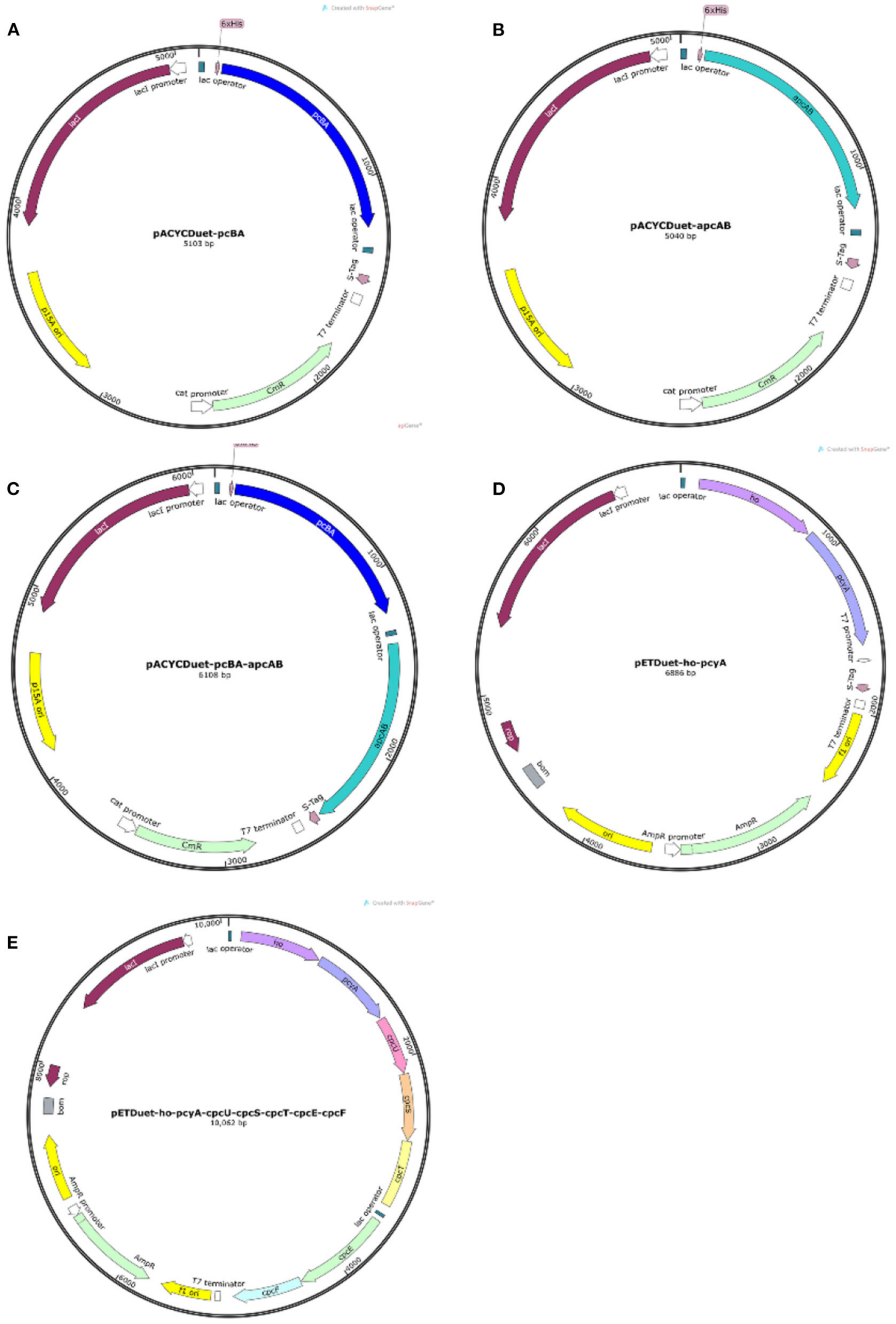
Plasmid map. **(A)** The plasmid pACYCDuet-*pcBA*; **(B)** the plasmids pACYCDuet-*apcAB*; **(C)** the plasmid pACYCDuet-*pcBA*-*apcAB*; **(D)** the plasmid pETDuet-*ho*-*pcyA*; **(E)** the plasmid pETDuet-*ho*-*pcyA*-*cpcU*-*cpcS*-*cpcT*-*cpcE*-*cpcF*.

The genes were respectively inserted into the vectors by enzyme digestion and ligation to obtain five kinds of plasmids, including pACYCDuet-*pcBA*, pACYCDuet-*apcAB*, pACYCDuet-*pcBA-apcAB*, pETDuet-*ho-pcyA*, and pETDuet-*ho-pcyA-cpcU-cpcS-cpcT-cpcE-cpcF* as shown in Figure 1. In the design of plasmid construction, the histidine tags in the plasmids pACYCDuet-*pcBA* and pACYCDuet-*pcBA* are located at the 5'-end of the *phycocyanin* gene and the *allophycocyanin* gene, which is convenient for subsequent protein purification. In the plasmid pACYCDuet-*pcBA*-*apcAB*, only the gene *pcBA* has a histidine tag in front of it. It can be used not only for subsequent protein purification, but also to detect whether the purified protein has a complex of phycocyanin and allophycocyanin, to analyze whether the recombinantly expressed phycocyanin and allophycocyanin can self-assemble.

### 3.2. Polyacrylamide gel electrophoresis and Western blot analysis for recombinant proteins

**Figure 2:**
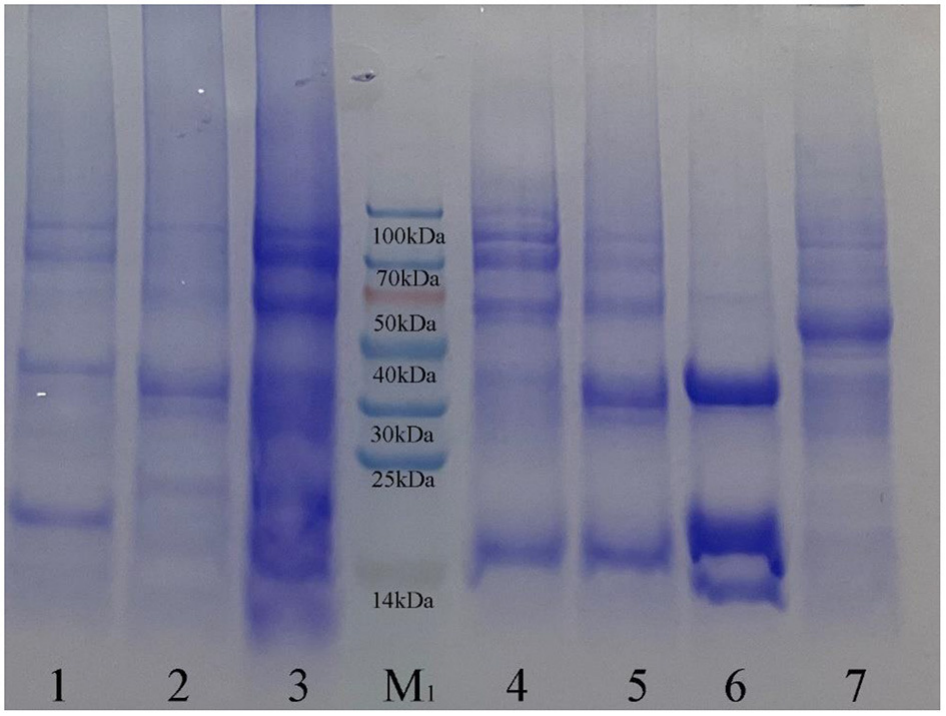
Sodium dodecyl sulfate polyacrylamide gel electrophoresis results. Lane1, Purified sample of target protein from strain *E. coli* PC; Lane2, Purified sample of target protein from strain *E. coli* APC; Lane3, Purified sample of target protein from strain *E. coli* PC-APC; Lane4, Purified sample of target protein from strain *E. coli* PC-APC-HPUSTEF Protein purification sample; Lane5, Purified sample of target protein from strain *E. coli* PC-HPUSTEF; Lane6, Purified sample of target protein from strain *E. coli* APC-HPUSTEF; Lane7, Unpurified protein sample of control *E. coli* BL21; Lane M_1_, SDS-PAGE Protein Marker.

**Figure 3:**
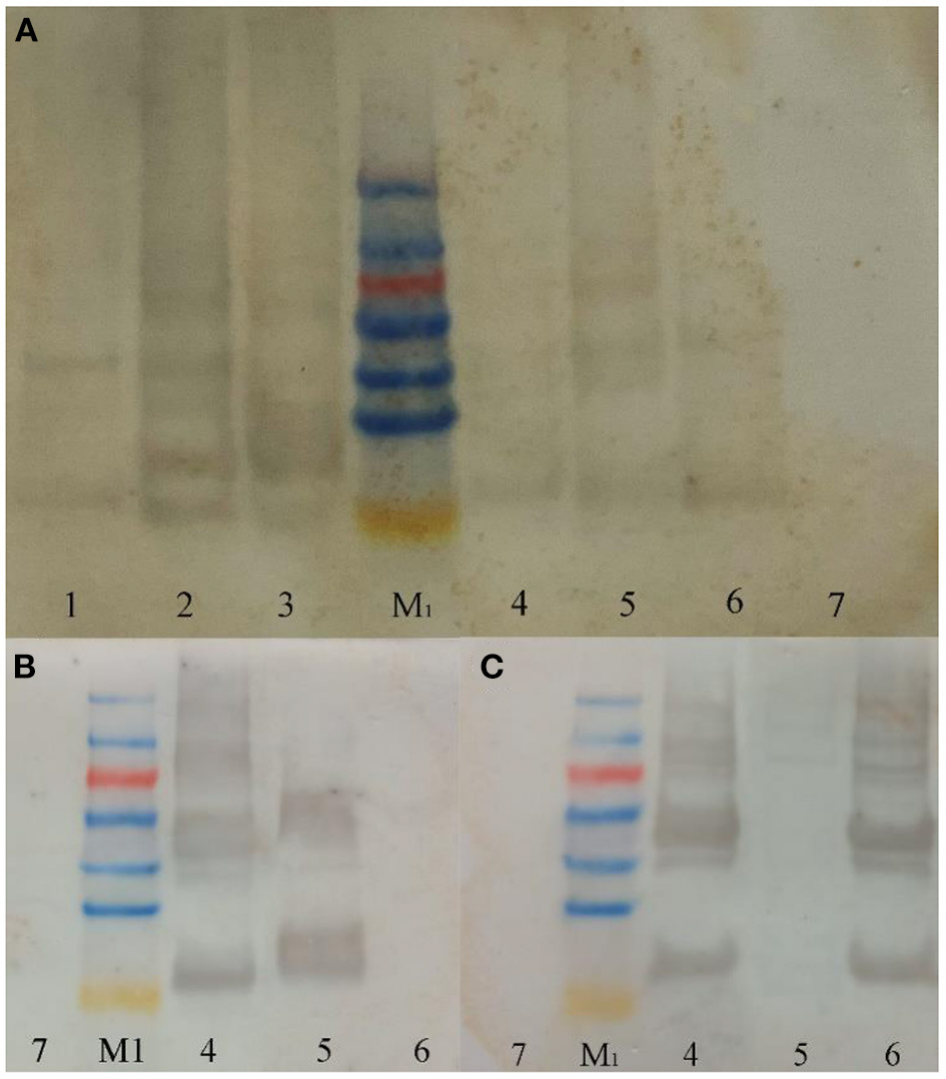
Western blot results. **(A)** Western-blot with Anti-6 × His Tag mouse monoclonal antibody; **(B)** western-blot with PC antibody; **(C)** western-blot with APC antibody. Lane1, Purified sample of target protein from strain *E. coli* PC; Lane2, Purified sample of target protein from strain *E. coli* APC; Lane3, Purified sample of target protein from strain *E. coli* PC-APC; Lane4, Purified sample of target protein from strain *E. coli* PC-APC-HPUSTEF Protein purification sample; Lane5, Purified sample of target protein from strain *E. coli* PC-HPUSTEF; Lane6, Purified sample of target protein from strain *E. coli* APC-HPUSTEF; Lane7, Unpurified protein sample of control *E. coli* BL21; Lane M_1_, SDS-PAGE Protein Marker.

From the detection results of SDS-PAGE (Figure 2) and Western blot (Figure 3), purified co-expressed recombinant phycocyanin-allophycocyanin samples have different molecular weight bands at about 14 kDa (single subunit), 30–40 kDa, 50–70 kDa, and 70–100 kDa, and the color of the bands from top to bottom deepen gradually. For the purified recombinant phycocyanin and recombinant allophycocyanin, the band is mainly at 30-−40 kDa. The size of the natural phycocyanin α subunit is about 17.6 kDa, the β subunit is about 18.05 kDa, and the molecular weight of the monomer (αβ) is about 35.65 kDa. The α subunit of natural allophycocyanin is about 17.39 kDa, the β subunit is about 17.33 kDa, and the monomer (αβ) is about 34.72 kDa. There are deep hybridization bands between 30 and 40 kDa. It is speculated that the heterologously expressed phycocyanin and allophycocyanin are mainly in the form of heterodimer (αβ), or some protein polymers may be depolymerized into heterodimers (αβ). In Western blot detection with specific antibody (Figures 3B, C), significant hybridization bands appeared at 30–40 kDa, 50–70 kDa, and 70–100 kDa, and the appearance of large molecular weight proteins indicated that there might be protein multimers produced in the recombinant strains, especially in the phycocyanin-allophycocyanin co-expression strain.

**Figure 4:**
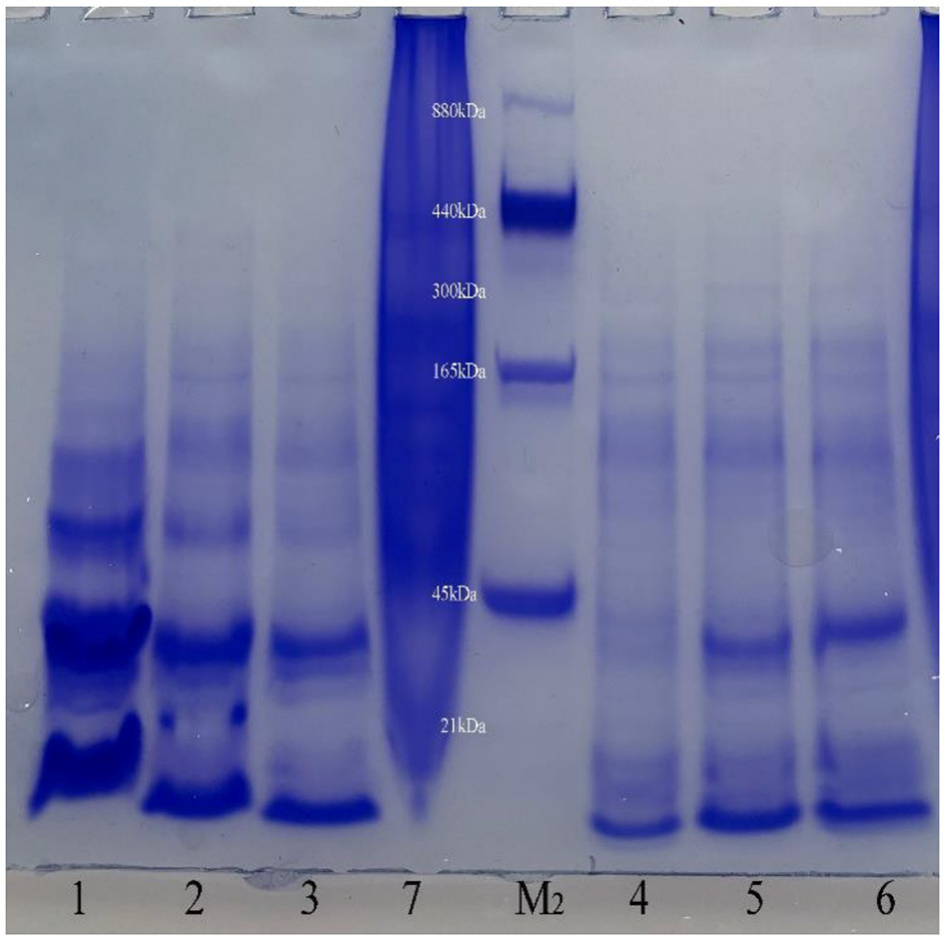
Native Polyacrylamide gel electrophoresis results. Lane1, Purified sample of target protein from strain *E. coli* PC; Lane2, Purified sample of target protein from strain *E. coli* APC; Lane3, Purified sample of target protein from strain *E. coli* PC-APC; Lane4, Purified sample of target protein from strain *E. coli* PC-APC-HPUSTEF Protein purification sample; Lane5, Purified sample of target protein from strain *E. coli* PC-HPUSTEF; Lane6, Purified sample of target protein from strain *E. coli* APC-HPUSTEF; Lane7, Unpurified protein sample of control *E. coli* BL21; Lane M_2_, Native-PAGE Protein Marker.

This conclusion was verified in Native PAGE (Figure 4). It can be seen from the figure that there are obvious bands of different molecular weights below 440 kDa in the six recombinant protein samples, indicating that the recombinant phycobiliprotein multimer exists. Like the results of SDS-PAGE, the concentration increases from top to bottom, which means that the recombinant proteins may have different combinations of sizes in *E. coli*. The formation of polymers with large molecular weight is relatively complicated, so the concentration is low; or due to no anchor of linker proteins, the structure of the polymers with large molecular weight is relatively loose and easy to depolymerize. Therefore, complexes of different molecular weights are produced during the depolymerization process, among which the concentration of phycocyanin and allophycocyanin in monomeric form is the highest. It can be seen from the figure that the clearly visible band with the largest molecular weight mainly exists around 300 kDa, and it is speculated that this may be the largest form of recombinant phycocyanin and allophycocyanin aggregates. In addition, the band between 45 and 165 kDa has the highest content in the large molecular weight proteins. It is speculated that the protein multimer here may be the easiest to form or the most stable polymer form.

### 3.3. Mass spectrometric detection

According to the analysis of Native-PAGE results, mass spectrometry detection and data analysis were performed on the 66 and 300 kDa protein samples of recombinant co-expressed phycocyanin-allophycocyanin on Native-PAGE. The detection results of LC-MS/MS showed that the two bands contained phycocyanin α, β subunits and allophycocyanin α, β subunits, indicating that recombinant allophycocyanin can combine with recombinant phycocyanin to form a high molecular weight protein complex (Supplementary Figures S1, S2). In the process of constructing the co-expression strains of phycocyanin and allophycocyanin, we only combined the histidine tag with phycocyanin, so if phycocyanin and allophycocyanin are detected in the purified sample, it indicates recombinant co-expressed allophycocyanin and phycocyanin can self-assemble and be purified by binding the histidine tag on the phycocyanin to Ni-NTA.

The theoretical size of the dimer (αβ)_2_ of allophycocyanin and phycocyanin is 70.361 kDa, which is basically consistent with the size of the band near 66 kDa, so the protein aggregate here is likely to be a dimer (αβ)_2_ [including phycocyanin monomer (αβ) and allophycocyanin monomer (αβ)]. It can also be inferred that the target bands of recombinant strains *E. coli* PC-HPUSTE and *E. coli* APC-HPUSTE around 66 kDa may be phycocyanin dimers (αβ)_2_ and allophycocyanin dimers (αβ)_2_ respectively. These dimers (αβ)_2_ are relatively stable in recombinant protein aggregates. Natural phycocyanin usually exists in the form of hexamers, and the theoretical molecular weight is about 213.864 kDa without linking polypeptides. Natural allophycocyanin usually exists in the form of a trimer, and the theoretical molecular weight is about 104.151 kDa without linking polypeptide. Then the molecular weight of the protein polymer combined with phycocyanin hexamer and allophycocyanin trimer is about 318.015 kDa, which is basically consistent with the target band size around 300 kDa, indicating that the protein polymer may be composed of phycocyanin hexamers and allophycocyanin trimers.

### 3.4. Fluorescence emission spectra

**Figure 5:**
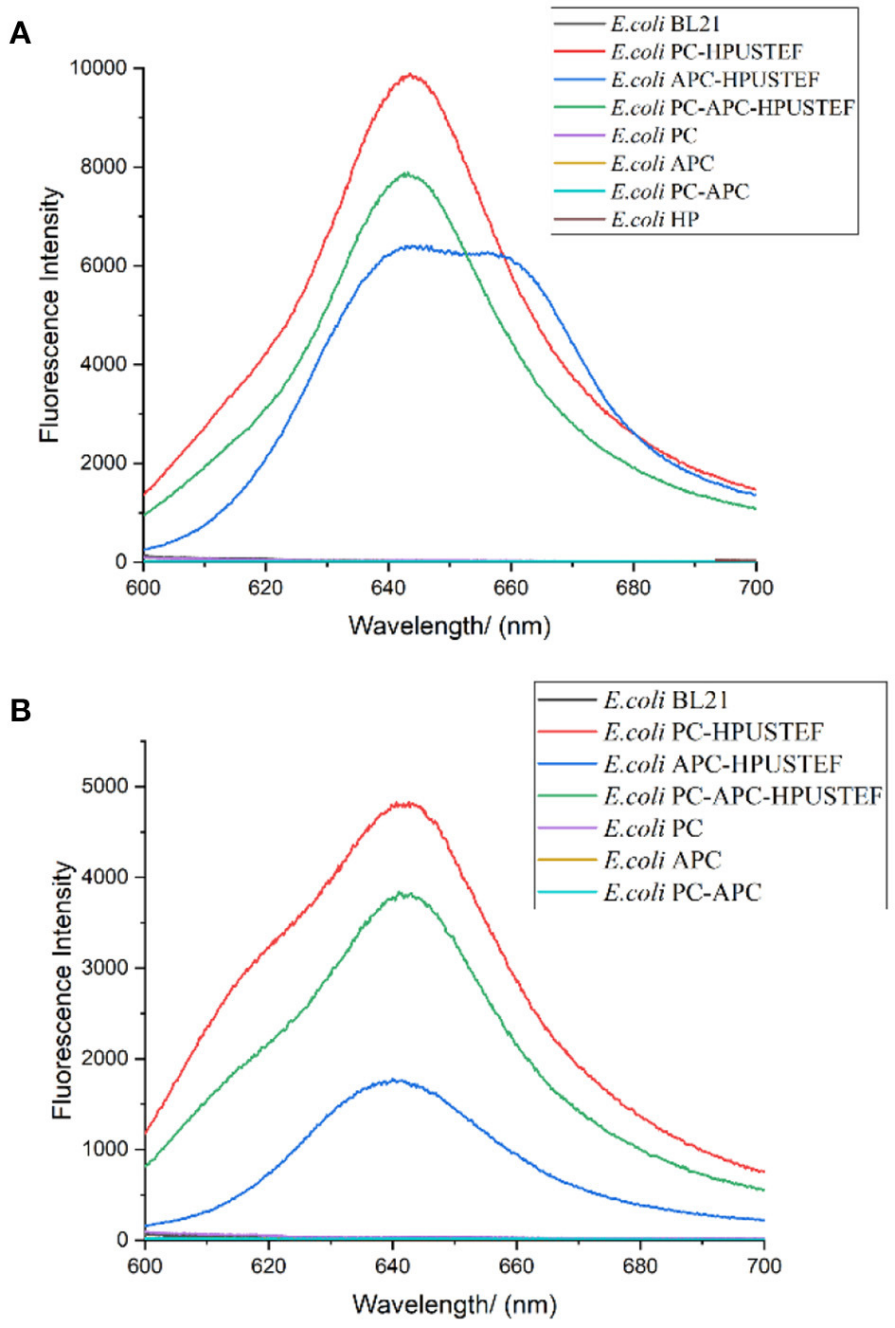
Fluorescence emission spectrum of recombinant strain (Excitation wavelength is 580 nm). **(A)** Unpurified protein samples of recombinant strain; **(B)** his-tag purified protein samples of recombinant strain.

In order to explore the optical properties of recombinant phycocyanin and allophycocyanin, recombinant phycocyanin (640 nm), recombinant allophycocyanin (650 nm/640 nm), recombinant phycocyanin- allophycocyanin (640 nm/650 nm) was scanned by fluorescence excitation spectrum, and it was found that the excitation spectrum peaks of the three recombinant phycobiliproteins were all located at about 580 nm, so the recombinant protein samples were excited at a wavelength of 580 nm to obtain the fluorescence emission spectra of different samples (Figure 5). It can be seen from the results (Figure 5A) that the recombinant protein co-expressed with the chromophore has high fluorescence activity, and the protein existing alone has no fluorescence activity. The fluorescence peak of recombinant strain *E. coli* PC-HPUSTEF is at 643 nm, which is the same as the peak position of natural phycocyanin. The peak position of *E. coli* APC-HPUSTEF is in the range of 640–660 nm, and the fluorescence emission peak of *E. coli* PC-APC-HPUSTEF is at about 640 nm.

According to the literature, fluorescence scanning of recombinant allophycocyanin in *E. coli* showed the fluorescence emission peak of both trimer and monomer was at around 660 nm (36). The emission wavelength of the allophycocyanin single subunit is about 640 nm (37, 38). In this paper, the peak position of *E. Coli* APC-HPUSTEF is in the range of 640–660 nm, which means that there are allophycocyanins with different polymerization forms in the sample.

Figure 5B is the fluorescence emission spectrum of the purified recombinant protein sample excited with a wavelength of 580 nm. The results show that the recombinantly expressed phycocyanin and allophycocyanin have fluorescence activity, and the apo-phycobiliprotein has no fluorescence activity. The fluorescence peaks of protein sample purified from *E. coli* PC-HPUSTEF were mainly concentrated at 640 nm. The fluorescence activity of phycocyanin is the highest, about 1.3 times that of recombinant phycocyanin-allophycocyanin, and 2.8 times that of recombinant allophycocyanin, while the fluorescence activity of holo-allophycocyanin is relatively low and locates at about 642 nm, and the fluorescence activity of the co-expressed phycocyanin-allophycocyanin is between phycocyanin and allophycocyanin, the fluorescence peak is located at 640 nm. The fluorescence peak of the protein is different from that before purification, which may be related to the different structure of the protein that can be purified.

### 3.5. Antioxidant capacity analysis

#### 3.5.1. Total antioxidant capacity

**Figure 6:**
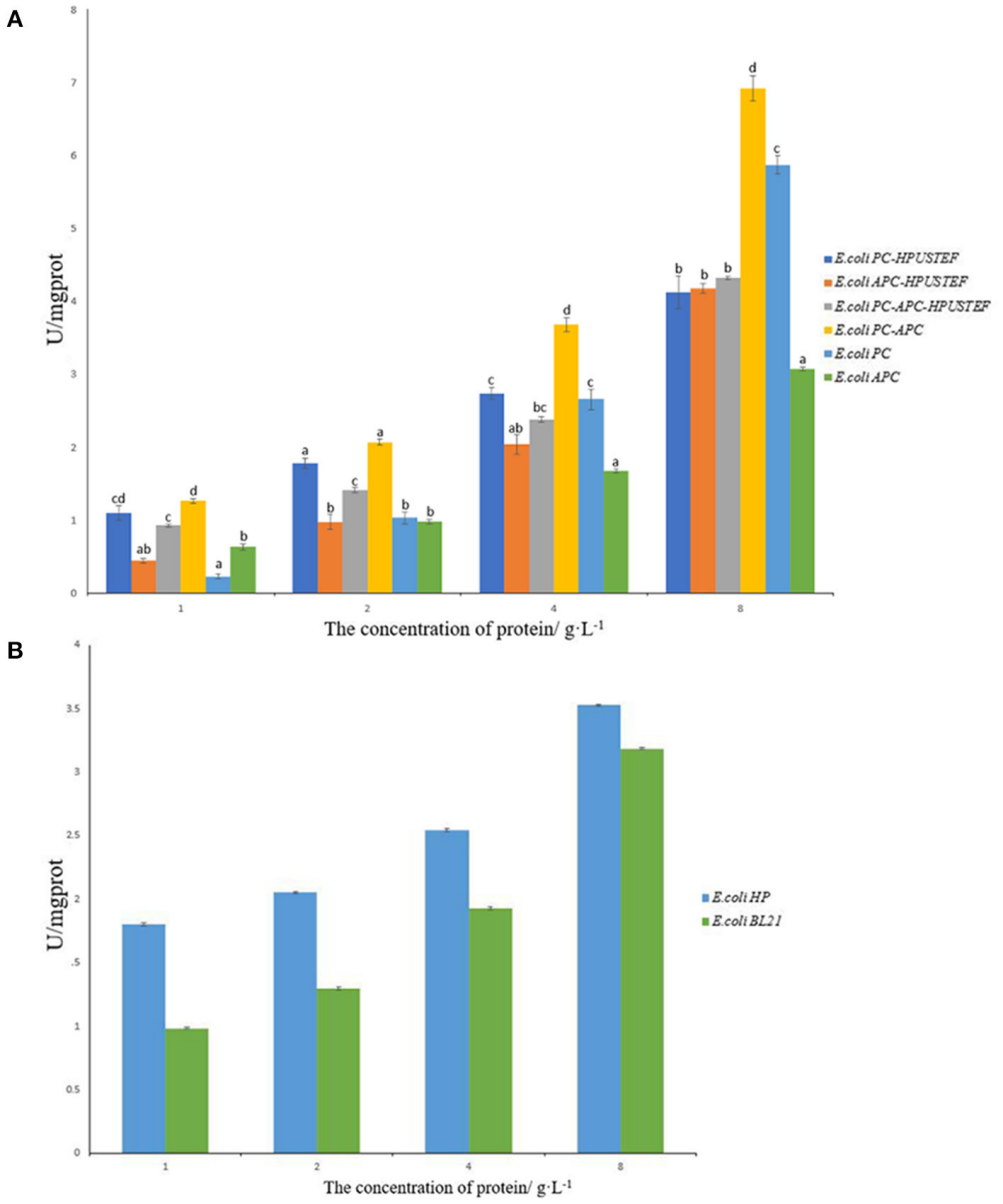
T-AOC total antioxidant capacity test results. **(A)** Antioxidant capacity of purified recombinant protein samples; **(B)** antioxidant capacity of *E. coli* HP, *E. coli* BL21 unpurified samples.

The total antioxidant capacity of the purified recombinant phycocyanin and allophycocyanin was tested. It can be seen from Figure 6 that the target products of the seven recombinant strains all exhibit antioxidant activity. Overall, the antioxidant activity of all recombinant proteins increased with increasing protein concentration (Figure 6A).

For the phycocyanin-allophycocyanin co-expression sample, the antioxidative activity of the target protein without fluorescence activity expressed by the strain *E. coli* PC-APC is higher than that with fluorescence activity, and the non-fluorescent phycocyanin-allophycocyanin had the highest antioxidant activity at different concentrations (*p* < 0.05).

For phycocyanin samples, when the protein concentration is 1 and 2 g/L, the antioxidant activity of phycocyanin with fluorescent activity is higher than apo-phycocyanin (*p* < 0.05). When the concentration is 4 g/L, there was no significant difference in the antioxidant activity of the two phycocyanin samples (*p* > 0.05). When the protein concentration reached 8 g/L, the activity of apo-phycocyanin was significantly higher than the phycocyanin sample with fluorescent activity (*p* < 0.05). For allophycocyanin samples, it can be seen from Figure 6A that when the concentration is 4 g/L and below, there is no significant difference in antioxidant activity between samples with and without fluorescence activity (*p* > 0.05). When the protein concentration reaches 8 g/L, allophycocyanin with fluorescent activity has strong antioxidant activity. In general, the antioxidant activity of all allophycocyanin samples was lower than that of phycocyanin and phycocyanin-allophycocyanin. Compared with antioxidant capacity of the samples with the concentration of 8 g/L, recombinant phycocyanin-allophycocyanin polymer has stronger T-AOC of 6.9 U/mgprot, which is about 1.17–2.25 times that of the other five proteins.

From Figure 6B, it can be seen that phycocyanobilin also has antioxidant activity. After combining with phycobiliprotein, it can show the enhancement effect of antioxidant activity at low concentration. But with the total sample concentration increases, the enhancement effect on the antioxidant activity of phycobiliprotein gradually becomes insignificant. This indicates that apo-phycobiliproteins is the main factor for its antioxidant activity. Phycocyanobilin can enhance the antioxidant activity of phycobiliprotein in a certain range, but the promotion effect is limited.

#### 3.5.2. DPPH free radical scavenging ability

**Figure 7:**
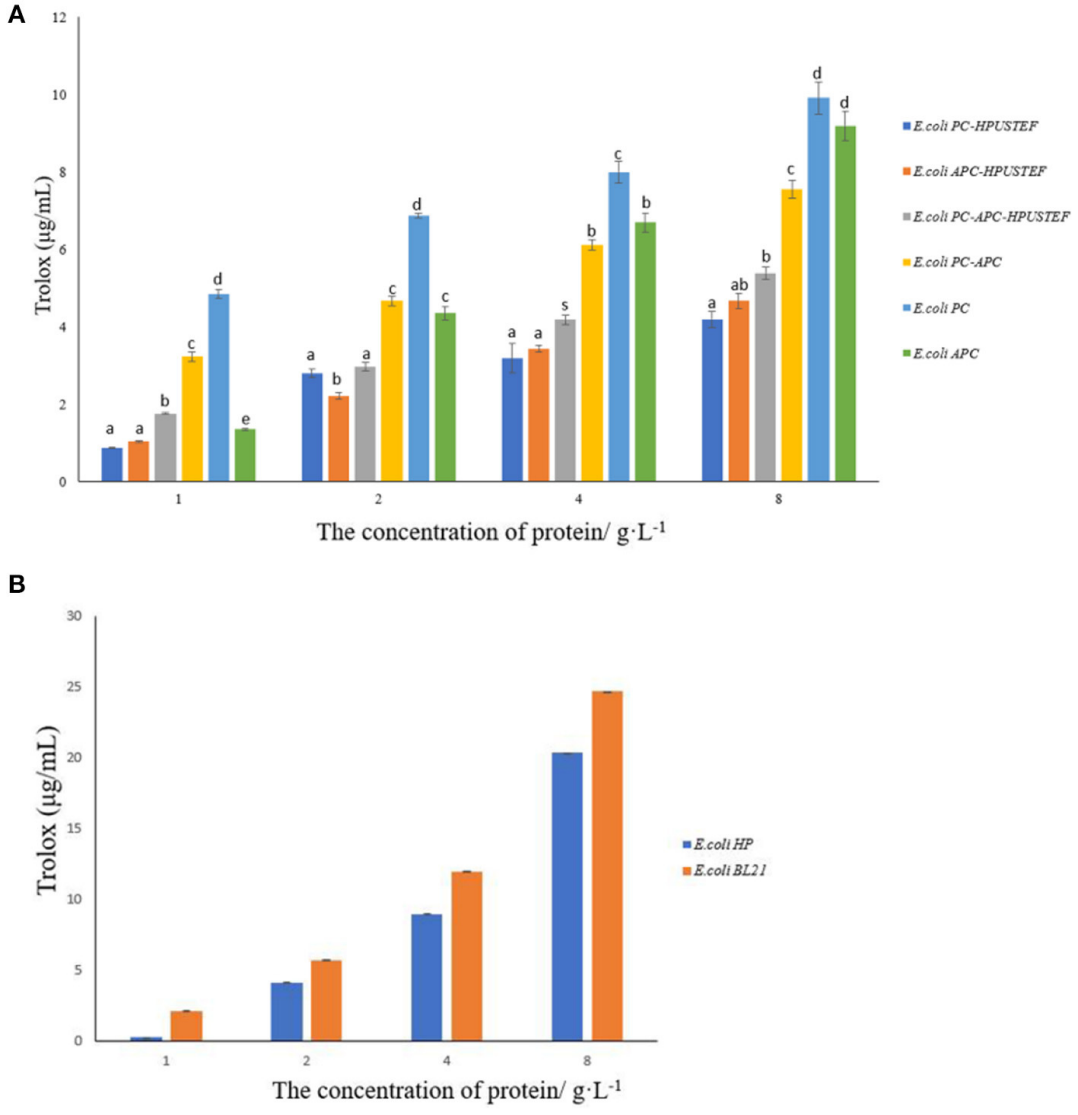
DPPH free radical scavenging ability results. **(A)** Antioxidant capacity of purified recombinant protein samples; **(B)** antioxidant capacity of *E. coli* HP, *E. coli* BL21 unpurified samples.

The results showed that the target proteins of the six purified recombinant strains all had the ability to scavenge DPPH free radicals (Figure 7A). Among them, the recombinant apo-phycocyanin has the strongest scavenging ability (*p* < 0.05), and the recombinant allophycocyanin with fluorescent activity has the weakest ability (*p* < 0.05). Compared with the antioxidant capacity of the samples with the protein concentration of 8 g/L, the recombinant phycocyanin has stronger DPPH antioxidant activity, which is about 1.2–2.5 times that of the other five proteins. Overall, the apo-phycobiliprotein samples were significantly higher than those of the three protein samples with fluorescent activity (*p* < 0.05), which indicated that the attachment of phycocyanobilin played a negative role in the ability of the recombinant phycobiliprotein to scavenge free radicals. As for the DPPH free radical scavenging ability test results of the chromophore (Figure 7B), the free radical scavenging ability of phycocyanobilin not combined with phycobiliprotein is lower than that of the control *E. coli* BL21, which indicates that the chromophore alone does not have the ability to provide hydrogen atom to scavenge DPPH free radicals. This phenomenon has the most obvious impact on phycocyanin, and the scavenging capacity of phycocyanin bound to chromophores has been reduced by about 60%.

In addition, the antioxidant capacity is related to the protein concentration. When the recombinant protein is at a low concentration (1 g/L), the ability of apo-allophycocyanin to scavenge free radicals is significantly lower than that of apo-phycocyanin and apo-phycocyanin-allophycocyanin polymer (*p* < 0.05). When the protein concentration reaches 2-−4 g/L, the scavenging ability of apo-allophycocyanin is enhanced to no significant difference from apo-phycocyanin-allophycocyanin polymer (*p* > 0.05). Especially when the concentration reaches 8 g/L, the free radical scavenging ability of apo-allophycocyanin is almost equivalent to that of apo-phycocyanin.

## 4. Discussion

In this paper, phycocyanin gene *pcBA*, allophycocyanin gene *apcBA*, heme oxidase gene *ho*, ferredoxin reductase gene *pcyA* and five chromophores Lyase genes *cpcU, cpcS, cpcT, cpcE*, and *cpcF* cloned from *Arthrospira platensis* FACHB314 were used to construct seven recombinant strains of *E. coli*. Three of them, including *E. coli* PC-APC, *E. coli* PC, and *E. coli* APC, expressed apo-phycobiliprotein without phycocyanobilin. Three strains, including *E. coli* PC-APC-HPUSTEF, *E. coli* PC-HPUSTEF, and *E. coli* APC-HPUSTEF could express holo-phycobiliprotein combined with phycocyanobilin. And the last one was *E. coli* HP, which only expressed phycocyanobilin. PAGE and Western blot were used to display the expression and assembly of phycobiliprotein. Fluorescence and antioxidant tests were carried out to verify the role of apo-phycobiliprotein and phycocyanobilin.

The difference between the co-recombination expression of phycocyanin-allophycocyanin and the recombination phycocyanin, recombination allophycocyanin was explored, as well as the possible protein aggregates formed by the heterologous combination of phycocyanin and allophycocyanin. The results of SDS-PAGE and Western Blot showed that all the recombinant strains successfully expressed phycocyanin and allophycocyanin. The Native PAGE results showed that obvious macromolecular protein bands appeared in the phycocyanin-allophycocyanin co-expression strains. Through mass spectrometry detection and protein amino acid sequence comparison, the results showed that the two protein aggregates were composed of phycocyanin and allophycocyanin. According to the molecular weight, the protein aggregate of about 66 kDa may be a dimer (αβ)_2_, which was composed of phycocyanin heterodimer (αβ) and allophycocyanin heterodimer (αβ). The protein aggregate of about 300 kDa may be the combined product of phycocyanin hexamer and allophycocyanin trimer. In *Arthrospira*, phycobiliproteins usually need to be linked by linker proteins to form high molecular polymers, forming a unified whole in light energy absorption, transmission, and biological activity (39). In this study, we found that recombinant phycocyanin and allophycocyanin can also spontaneously combine into large molecular weight polymers, probably because there are similar segments in the amino acid sequences of phycocyanin and allophycocyanin. This laid the foundation for studying the activity and function of phycocyanin and allophycocyanin polymers.

By scanning the fluorescence spectrum, the fluorescence peak of recombinant strain *E. coli* PC-HPUSTEF is at 643 nm, which is similar to the peak position of natural phycocyanin. The peak position of *E. coli* APC-HPUSTEF is in the range of 640–660 nm, while the fluorescence emission peak of natural allophycocyanin is about 650 nm. According to the literature, fluorescence scanning of recombinant allophycocyanin in *E. coli* showed the fluorescence emission peak of both trimer and monomer was at around 660 nm (36). The emission wavelength of the allophycocyanin single subunit is about 640 nm (37, 38). In this paper, the peak position of *E. Coli* APC-HPUSTEF is in the range of 640–660 nm, which means that there are allophycocyanins with different molecular weights polymerization forms in the sample. After the protein is purified, the fluorescence peak changes. The fluorescence peak of *E. coli* PC-HPUSTEF purified protein sample is mainly concentrated at 640 nm, which is also the same as the natural phycocyanin. There is a small shoulder peak at 620 nm, which may be the existence of some other structural intermediates, or because of the structure of the recombinant protein different from natural phycocyanin. The fluorescence peak of the purified recombinant protein of strain E. coli APC-HPUSTEF is located at about 642 nm, which is different from the fluorescence characteristic peak of natural allophycocyanin at 650 nm, and the fluorescence peak of the unpurified protein sample is at 640–660 nm. The reason may be that in the different assembly structures formed by recombinant allophycocyanin. In addition, the chromophore is formed by connecting four pyrrole rings, which are A ring, B ring, C ring, and D ring. The B ring and C ring of the chromophore are almost coplanar, and the increase of the dihedral angle of the A ring and D ring reduces the conjugation of the chromophore, which also causes a blue shift in the fluorescence emission spectrum (40). And also, the interactions of the aromatic amino acids of the linker proteins with the chromophores may be a key factor in fine-tuning the energy states of the chromophores to ensure the efficient unidirectional transfer of energy (39, 41). The recombinant protein expression system herein does not contain linker proteins. Therefore, the microenvironment of the chromophore may affect the position of the fluorescence emission peak of the recombinant proteins.

Through the detection of the fluorescence activity of the recombinant proteins, we found that the fluorescence activity of the recombinant phycocyanin is the highest, and the fluorescence peak position is concentrated (643 nm), which is beneficial to be used as a natural pigment and dye in the food industry, and as a fluorescent probe in the medical diagnosis. Natural allophycocyanin is blue-green, and its fluorescence peak position (640–660 nm) is different from that of phycocyanin (643 nm). It is also a potential substance for products such as pigments and dyes. However, due to its extremely similar properties to phycocyanin, it is very difficult to extract a large amount of purified allophycocyanin from algae. The general extraction method has low purity and less protein, and can only obtain about 50% of the total allophycocyanin. The use of higher process technology will result in a substantial increase in cost and higher selling price, which is not conducive to the application of downstream products. However, the recombinant allophycocyanin in this paper overcomes the difficulty of purification, and can obtain high-purity allophycocyanin with fluorescent activity simply and quickly. Recombinant phycocyanin-allophycocyanin polymer did not show more prominent fluorescence intensity and unique fluorescence peaks. Therefore, recombinant phycocyanin or allophycocyanin can meet general requirements in terms of being used as a fluorescent marker.

A number of studies have shown that the excessive production of free radicals or the decline in the body's ability to scavenge free radicals will lead to the occurrence and deterioration of various diseases (42–44). Therefore, scientists are constantly looking for substances and methods that can scavenge free radicals and reduce the harm of free radicals to the human body. Antioxidants can effectively overcome the damage caused by free radicals to human health, and natural antioxidants are more popular, so the market demand is huge. Natural phycobiliprotein is currently one of the most popular natural antioxidants (15), and the use of molecular biology and bioengineering techniques to recombine phycobiliprotein is an effective way to meet market demand. Therefore, the antioxidant detection of recombinant phycobiliprotein was carried out in this paper.

Many antioxidant substances can reduce ferric ions to ferrous ions. Therefore, total antioxidant capacity of recombinant phycobiliproteins were tested based on the principle of measuring iron reducing power. First, all recombinant phycobiliprotein samples had antioxidant activity. By comparing the results of recombinant apo-phycobiliprotein and recombinant holo-phycobiliprotein, it was found that chromophore also has antioxidant effect, but the effect is limited, and phycocyanobilin can only show the promotion effect of antioxidant activity at a moderate concentration. When the concentration of recombinant phycobiliprotein was 4 g/L or below, the activity of phycobiliprotein with fluorescent activity was stronger; and when the phycobiliprotein reached a high concentration (8 g/L), the role of chromophore was no longer significant. So it is speculated that the concentration of phycobiliproteins was the main factor affecting the antioxidant activity. This means that in the electron transfer-based antioxidant system, the protein part of phycobiliprotein mainly plays an antioxidant role, which is consistent with the MEI's research result (45), he showed that in the antioxidant system based on active oxygen, the main antioxidant group of phycocyanin is the protein part. Moreover, the fluorescence detection results of the recombinant strain *E. coli* HP expressing only phycocyanobilin showed that the antioxidant activity of phycocyanobilin alone was not strong, and with the increase of concentration, the antioxidant activity gradually became insignificant. This also shows that the reduction effect of phycocyanobilin and phycobiliprotein can be better after the combination of them, which may be because that the environment after the tetrapyrrole structure is covalently linked to phycobiliprotein can affect the antioxidant activity of phycocyanobilin. And also the different sizes of aggregates produced by recombinant phycocyanin-allophycocyanin may also affect or promote antioxidant activity to a certain extent by the assembly structure of the active site masking and exposure. Therefore, the co-expression of phycocyanin-allophycocyanin polymers has the highest antioxidant activity, which may also be due to this reason.

In addition, DPPH free radicals are often used as markers in scientific research to evaluate molecular hydrogen donating capacity and antioxidant activity. Antioxidant activity was determined by the odd-numbered electrons of the nitrogen atom in the stable radical DPPH being reduced to the corresponding hydrazine by receiving a hydrogen atom from the antioxidant (46). In the results of this study, the recombinant phycobiliprotein has the ability to scavenge DPPH free radicals, while the recombinant chromophore did not show the ability to scavenge free radicals, and the attachment of the chromophore even has a negative effect on the scavenging ability of the recombinant proteins. This result differs from Wu's results for recombinant PCB-CpcB and apo-CpcB. In his research, binding of phycocyanobilin to the phycocyanin β subunit enhanced the ability to scavenge free radicals (47). The reason may be that the recombinant phycobiliprotein α subunit and β subunit were co-expressed in *E. coli* in this research. So there are differences in the combination of subunits and the binding of chromophores and recombinant polymers. And also the microenvironment of chromophore binding in recombinant holo-PC/holo-APC is different. These may play an important role in antioxidation capacity. Estrada's research found that freezing, increasing, or decreasing pH value will lead to the change of the charge of amino acid residues around the phycobiliprotein chromophore, and also affect the interaction between chromophore and apo-phycobiliprotein (48). These factors will affect the ability of phycobiliproteins to scavenge free radicals, which indicates that the protein part around the chromophore of phycobiliproteins will affect its antioxidant capacity. Therefore, the contribution of both protein and chromophore components to the overall antioxidant properties of phycobiliproteins is controversial (46–48). However, combined with multiple studies (30–35, 39, 41, 45–50), it can be shown that no matter whether the chromophore is combined or not, the recombinant phycocyanin, recombinant allophycocyanin, and co-expressed phycocyanin-allophycocyanin polymer all have the ability to scavenge DPPH free radicals.

In summary, recombinant phycocyanin, allophycocyanin, and their polymers with fluorescent activity and antioxidant activity were successfully expressed in this study, which laid the foundation for their application in food, medicine, and other industries. From the results of this paper, in order to obtain related phycobiliprotein derivative products as the fluorescent probes or fluorescent markers, phycocyanin with chromophore is the best choice. For the purpose of eliminating free radicals, the above three proteins are potential anti-oxidation health products. Among them, in the anti-oxidation activities based on the removal of catalytic metal ions, such as food antioxidants or anti-corrosion, the recombinant phycocyanin-allophycocyanin co-expression polymer without fluorescent activity is more suitable. In the scavenging DPPH radicals that need to provide hydrogen atoms, the antioxidant activities of phycocyanin without fluorescence activity is the highest. In addition, if it needs to have fluorescent properties or be used as a colorant in the antioxidant application process, recombinant phycocyanin with fluorescent activity and recombinant phycocyanin-allophycocyanin polymers is a good choice.

## Data availability statement

The original contributions presented in the study are publicly available. This data has been deposited to the ProteomeXchange Consortium *via* the PRIDE partner repository with the dataset identifier PXD040154.

## Author contributions

X-nZ designed the research, involved in interpreting the results, and editing the manuscript. M-hS, YB, and X-tX conducted the research. M-hS, J-fS, and X-nZ analyzed the data. M-hS and X-nZ wrote the paper and had primary responsibility for the final content. All authors read and approved the final manuscript.
