# Supplementary material for: Fluorescence and antioxidant activity of heterologous expression of phycocyanin and allophycocyanin from Arthrospira platensis
Source: Front Nutr. 2023 Feb 20;10:1127422. doi: 10.3389/fnut.2023.1127422 (PMC9987159; doi:10.3389/fnut.2023.1127422)
Supplement: Supplementary file 1 [file Data_Sheet_1.PDF]

A

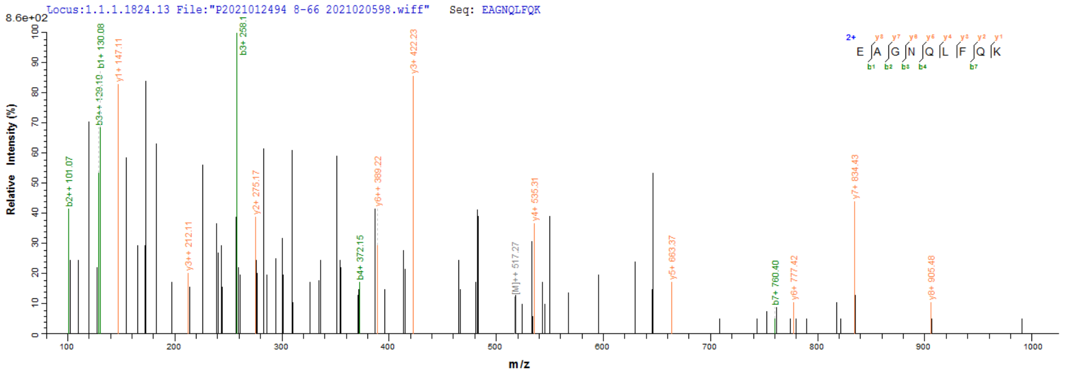
Locus:1.1.1.1824.13 File:"P2021012494 8-66 2021020598.wiff" Seq: EAGNQLFQK
8.6e+02
Relative Intensity (%)
m/z
E A G N Q L F Q K

B

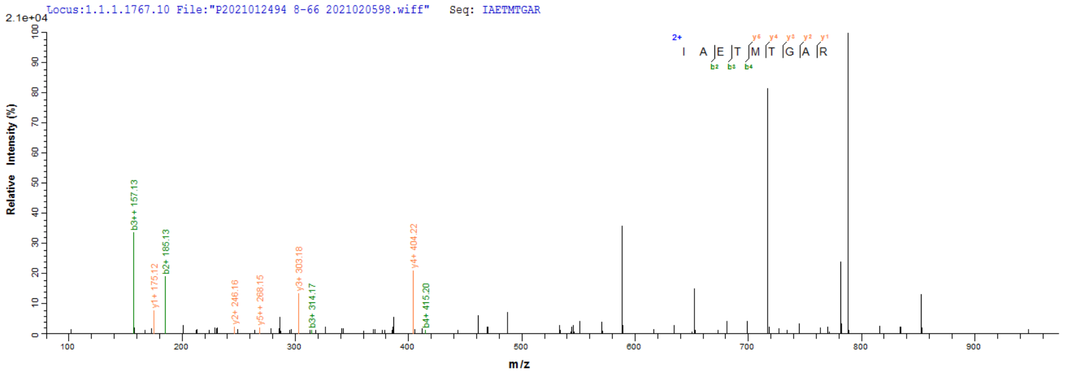
Locus:1.1.1.1767.10 File:"P2021012494 8-66 2021020598.wiff" Seq: IAETMTGAR
2.1e+04
Relative Intensity (%)
m/z
I A E T M T G A R

C

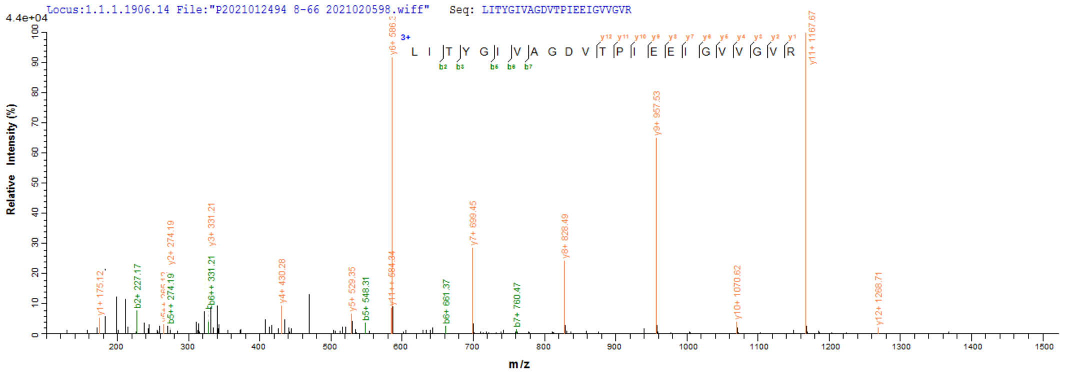
Locus:1.1.1.1906.14 File:"P2021012494 8-66 2021020598.wiff" Seq: LITYGIVAGDVTPIEEIGVVGVR
4.4e+04
Relative Intensity (%)
m/z
L I T Y G I V A G D V T P I E E I G V V G V R

D

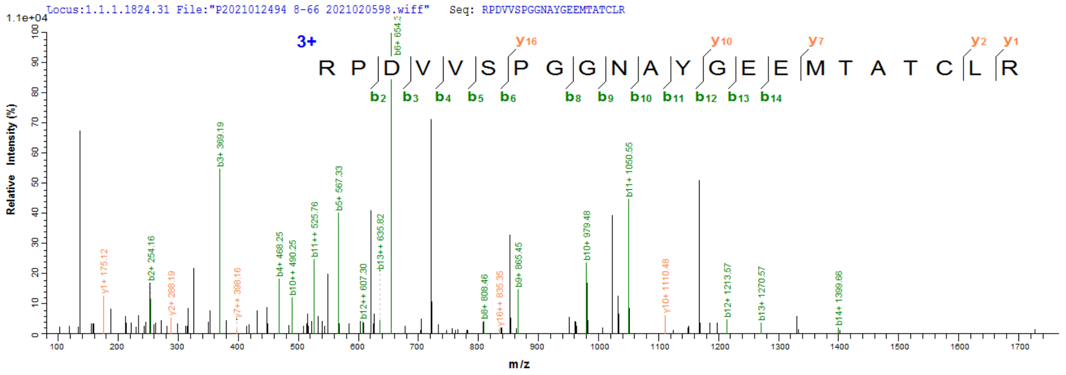
Locus:1.1.1.1824.31 File:"P2021012494 8-66 2021020598.wiff" Seq: RPDVVSPGGNAYGEEMTATCLR
1.1e+04
Relative Intensity (%)
m/z
3+
R P D V V S P G G N A Y G E E M T A T C L R

E

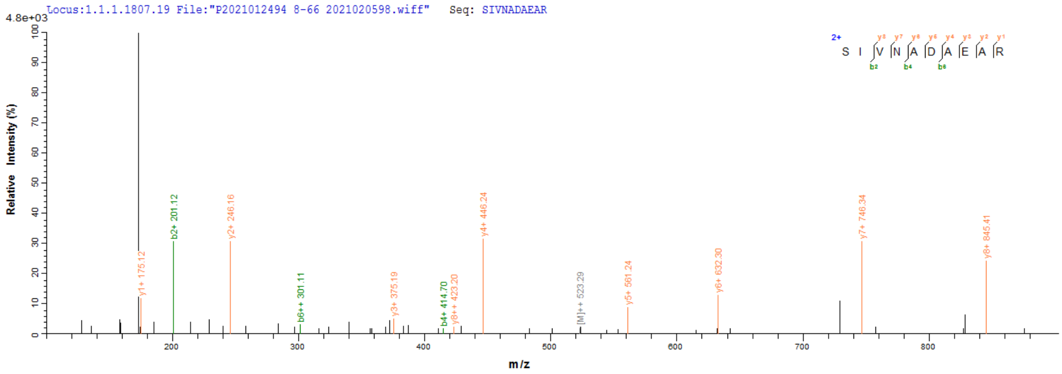

F

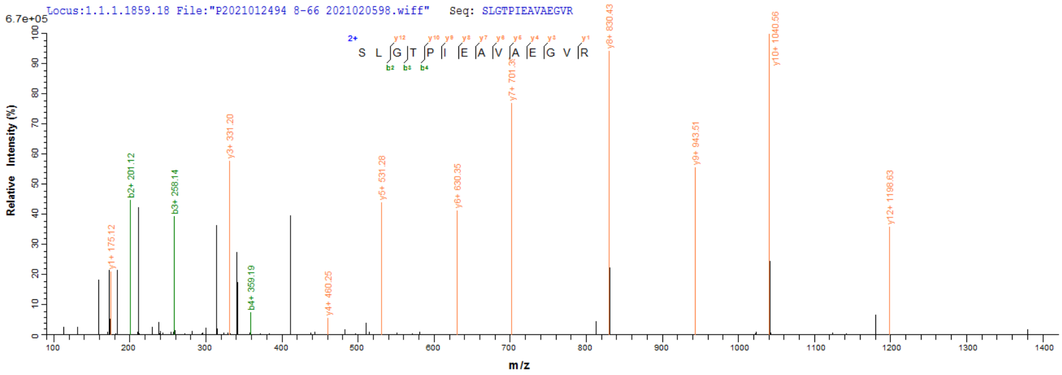

G

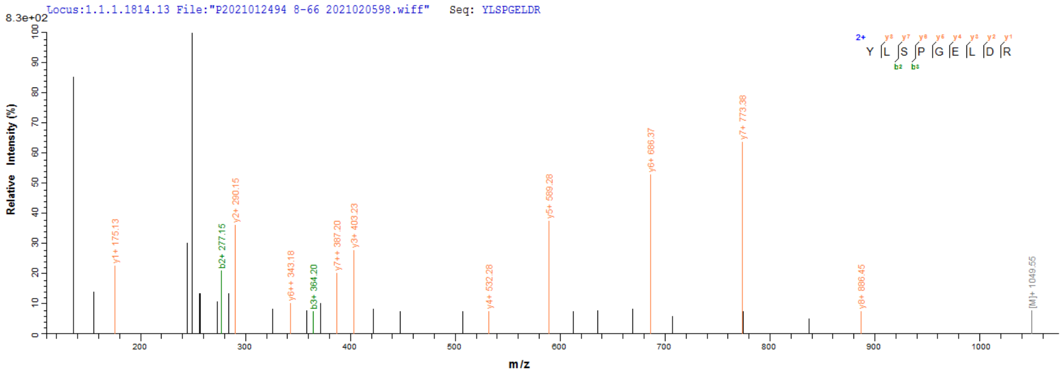

Figure S1 secondary mass spectrum of 66kDa protein band.

A

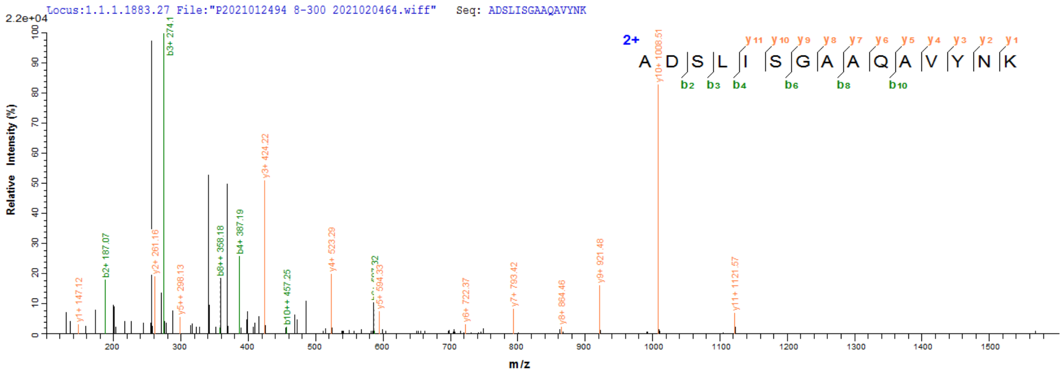
Locus:1.1.1.1883.27 File:"P2021012494 8-300 2021020464.wiff" Seq: ADSLISGAAQAVYNK
2.2e+04
Relative Intensity (%)
m/z
2+

B

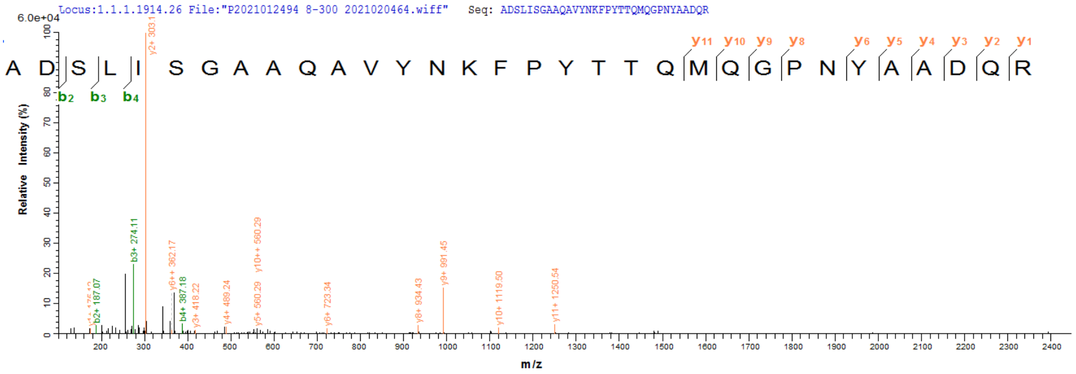
Locus:1.1.1.1914.26 File:"P2021012494 8-300 2021020464.wiff" Seq: ADSLISGAAQAVYNKFPYTTQMQGPNYAADQR
6.0e+04
Relative Intensity (%)
m/z

C

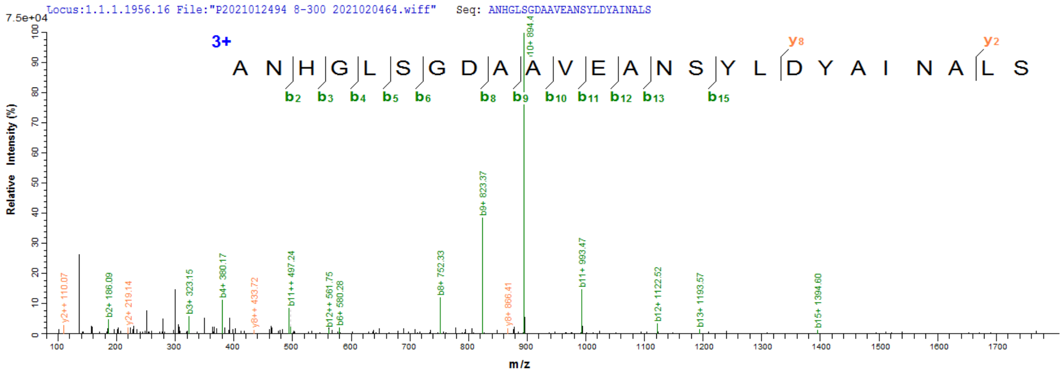
Locus:1.1.1.1956.16 File:"P2021012494 8-300 2021020464.wiff" Seq: ANHGLSGDAAVEANSYLDYAINALS
7.5e+04
Relative Intensity (%)
m/z
3+

D

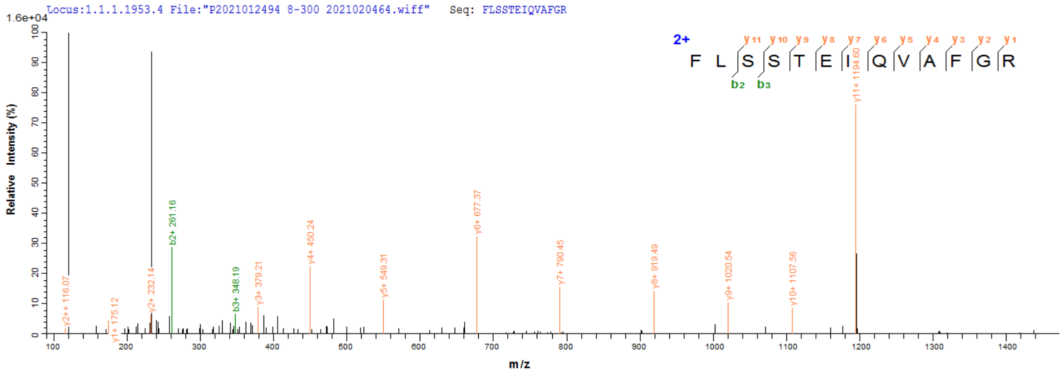
Locus:1.1.1.1953.4 File:"P2021012494 8-300 2021020464.wiff" Seq: FLSSTEIQVAFGR
1.6e+04
Relative Intensity (%)
m/z
2+

E

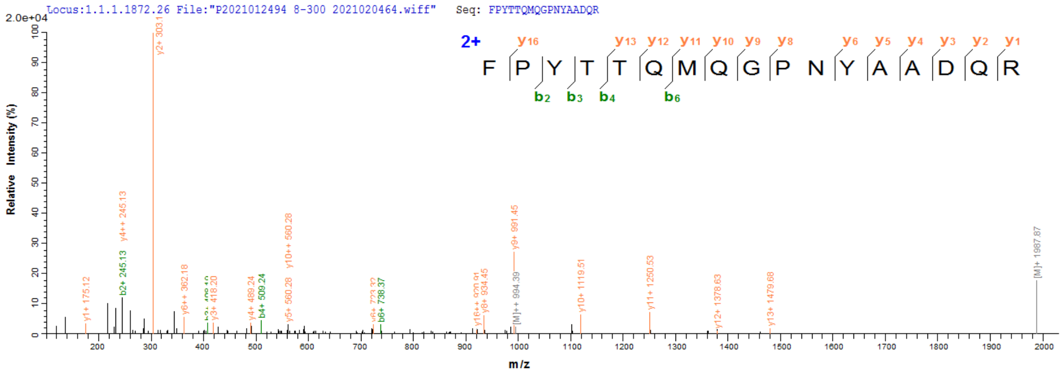

F

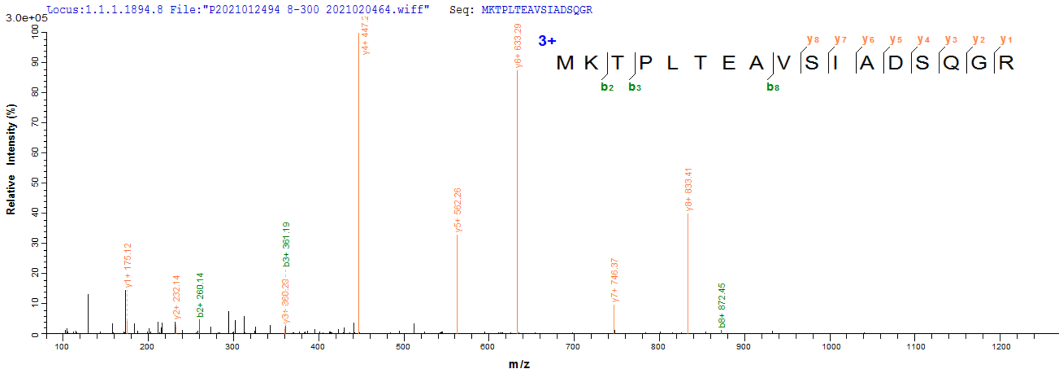

G

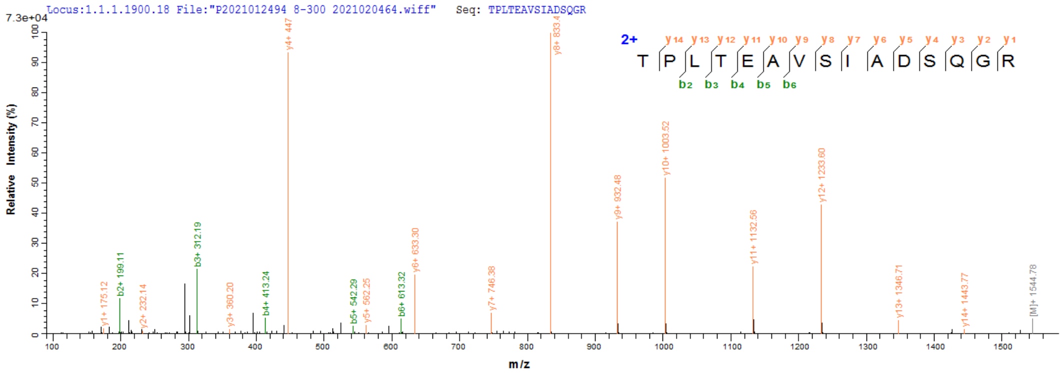

Figure S2 secondary mass spectrum of 300kDa protein band.
